# Adult-onset multifocal kaposiform hemangioendothelioma in the bone marrow, lung, liver, and brain: a case report

**DOI:** 10.3389/fonc.2024.1322684

**Published:** 2024-02-22

**Authors:** Alexa Bello, Mir B. Alikhan, Aparna Subramaniam, Zenab I. Yusuf, Bruce Brockstein, Vinod Ravi

**Affiliations:** ^1^ Department of Sarcoma Medical Oncology, The University of Texas MD Anderson Cancer Center, Houston, TX, United States; ^2^ Department of Pathology, Northshore University Health System, Evanston, IL, United States; ^3^ Division of Hematology / Oncology, Northshore University Health System, Evanston, IL, United States

**Keywords:** kaposiform hemangioendothelioma, adult, bone marrow, myelofibrosis, case report

## Abstract

Kaposiform hemangioendothelioma (KHE), a rare form of vascular neoplasm, is typically seen in children. In this paper, we report a unique case of KHE replacing bone marrow tissue mimicking myeloproliferative neoplasm with additional involvement in the lung, liver, and brain in a 60-year-old Caucasian woman. The patient was initially seen in the hematology department for the chief complaint of epigastric pain and anemia. Abdominal magnetic resonance imaging (MRI) revealed mild splenomegaly with iron deposition secondary to extramedullary hematopoiesis. Additional workup was inconclusive. Subsequent bone marrow and lung biopsies eventually revealed bone marrow with extensive grade 3 fibrosis and multiple foci of low-grade vasoformative neoplasm in the lung suggestive of KHE. Although rare, KHE can present as an aggressive disease with indolent behavior in adults and can be distinguished from other vascular malignancies based on histopathology and imaging findings.

## Introduction

Kaposiform hemangioendothelioma (KHE) is a rare, locally aggressive vascular neoplasm classically described in children (1). Reports in adults are rare, but have been described in the literature in approximately 26 cases (2–6). Its etiology remains unknown. Pathophysiological changes are due to secondary dysregulation of lymphangiogenesis and angiogenesis via VEGF-C/VEGFR3 and Ang-2/Tie-2 signaling pathways leading to downstream PI3K/Akt/mTOR signaling, mediated by PIK3CA (2, 7).

Clinically, KHE presents as a locally aggressive neoplasm of firm consistency with erythematous violet-colored plaques within the skin and adjoining subcutaneous tissue. It commonly affects the extremities, retroperitoneum, and the head and neck region (2, 8). Despite KHE being locally aggressive, very few rare cases of multifocal KHE have been reported in the literature (9–12). Biopsy remains the gold standard diagnostic test (7). Histologically, KHE is characterized by irregular tumor margins, infiltrating rounded nodules composed of endothelial cells of spindled morphology, and slit-like vascular channels (13, 14). Immunohistochemistry (IHC) shows positive staining for both lymphatic markers and vascular endothelial markers (4, 7, 15, 16). A major prognostic factor is the presence of the Kasabach–Merritt phenomenon (KMP), which is characterized by life-threatening consumptive coagulopathy and severe thrombocytopenia (16–18). Despite KMP being less common in adults (11%) compared to children (77%), it is present in up to 70% of KHE cases at the time of presentation (8, 19). To date, there is no consensus regarding an ideal treatment for KHE as the disease is rather rare, especially in adults (7, 13, 20). Surgery with wide margins of excision is often curative; however, it is challenging to successfully perform due to the infiltrative nature of the disease (2, 13, 21). For extensive or unresectable tumors, combined treatment strategies are recommended, particularly if accompanied by KMP (20). Based on the available literature, combination treatment with vincristine and corticosteroids, antifibrinolytic agents, systemic interferons, and mammalian target of rapamycin (mTOR) inhibitors; transarterial embolization; and radiation therapy have shown clinical benefits in KHE (2, 11, 16, 22). Previously, vincristine and corticosteroids have been recommended as the first-line treatment for KHE (23). However, in recent years, sirolimus, an mTOR inhibitor, has shown promising clinical benefits in patients with KHE (24, 25). Although few prospective studies assessing different treatment regimens have been reported in the literature, the assessment of these combined therapies in adult cases warrants further investigation (24, 26, 27). To the best of our knowledge, we hereby describe the first case of KHE involving the bone marrow, liver, lung, and brain in an adult.

## Case description

**Figure 1:**
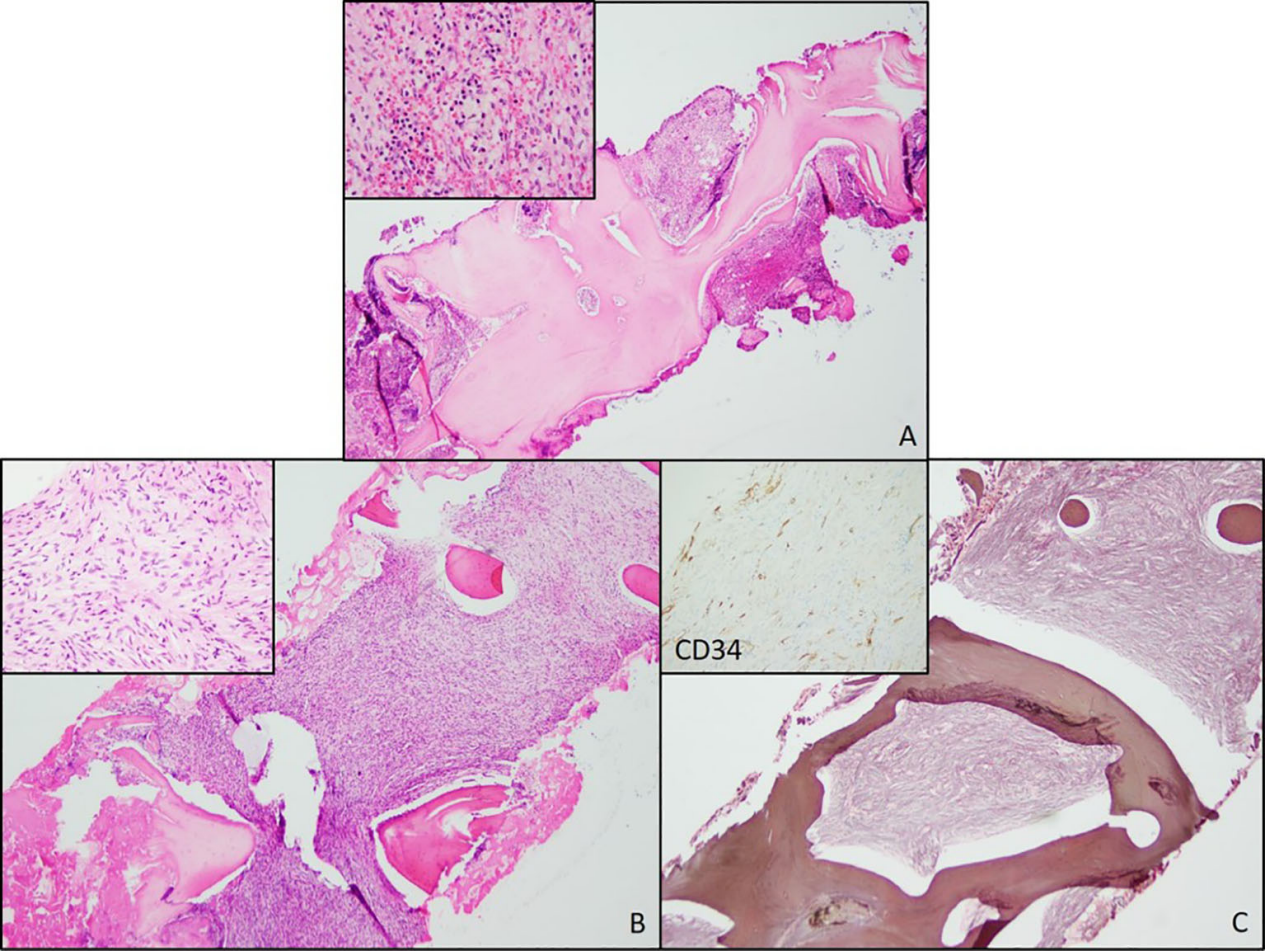
Bone Marrow Biopsies from 2018 and 2020. **(A)** Bone marrow biopsy from 2018 showing trabecular thickening and increased cellularity. **(B)** Bone marrow biopsy from 2020 also showing increased cellularity and increased numbers of atypical spindle cells (inset). **(C)** A reticulin stain showing extensive fibrosis. A negative CD34 immunostain in the atypical spindle cells (inset).

A 60-year-old Caucasian woman presented in April 2018 with back and epigastric pain. Medical history was relevant for chronic kidney disease and bilateral hydronephrosis status post-stent replacement. The initial physical examination showed normal results. Routine laboratory workup was only relevant for normocytic anemia [hemoglobin (Hb) = 10 g/dL, mean corpuscular volume (MCV) = 93.6]. Magnetic resonance imaging (MRI) showed mild splenomegaly with diffuse heterogeneous T1 and T2 hypointensity compatible with iron deposition secondary to extramedullary hematopoiesis. The initial peripheral blood smear was consistent with normocytic anemia. Bone marrow biopsy (BMBx) revealed normocellular marrow (40%) with osteosclerosis and focal paratrabecular fibrosis associated with increased mast cells without morphologic evidence of primary bone marrow pathology. IHC stains were positive for tryptase, CD117, and CD34 (**Figure 1A**). Serum tryptase levels and molecular studies were normal. The findings were nonspecific and inconclusive. Following this, our patient was assessed across multiple institutions with multidisciplinary workups that only revealed worsening anemia, new-onset hepatomegaly, and progressive replacement of bone marrow by fibrosis.

In August 2020, a routine laboratory workup showed worsened anemia (Hb = 6.9 g/dL), new macrocytosis (MCV = 107.4), and new mild thrombocythemia (122 × 10^3^/μL). BMBx showed 100% cellularity and an increased number of atypical spindle cells. Reticulin stain showed severe grade 3 myelofibrosis (MF3) (**Figures 1B, C**). Ancillary testing included molecular studies for myeloproliferative neoplasms (e.g., JAK2, CALR, and MPL) and mastocytosis (e.g., KIT), which were all negative. Bone density (DXA scan) and beta-glucosidase were assessed to rule out Gaucher’s disease and were found unremarkable. This BMBx was sent to two different institutions for additional expert consultation. Based on the collective assessment, this process was unlikely to be a myeloproliferative neoplasm and less unlikely to be a primary/metastatic mesenchymal neoplasm, but rather a presumptive case of autoimmune myelofibrosis. Empirical prednisone 1 mg/kg was recommended and started to determine whether this was in fact a case of autoimmune myelofibrosis. Additional autoimmune workups were unremarkable, and an unclear response to prednisone was observed. The patient’s hemoglobin levels improved from 6.9 to 9.0 g/dL during empirical prednisone administration. However, the patient had trouble tolerating it as she experienced symptoms of fatigue and weakness all along. Furthermore, due to unremarkable extensive bone metabolism, autoimmune workups, and the lack of substantial clinical benefit to corticosteroids, autoimmune myelofibrosis was ruled out and prednisone was ultimately tapered down to 5mg, with subsequent hemoglobin levels declining again to 6.4 g/dL at its lowest value.

**Figure 2:**
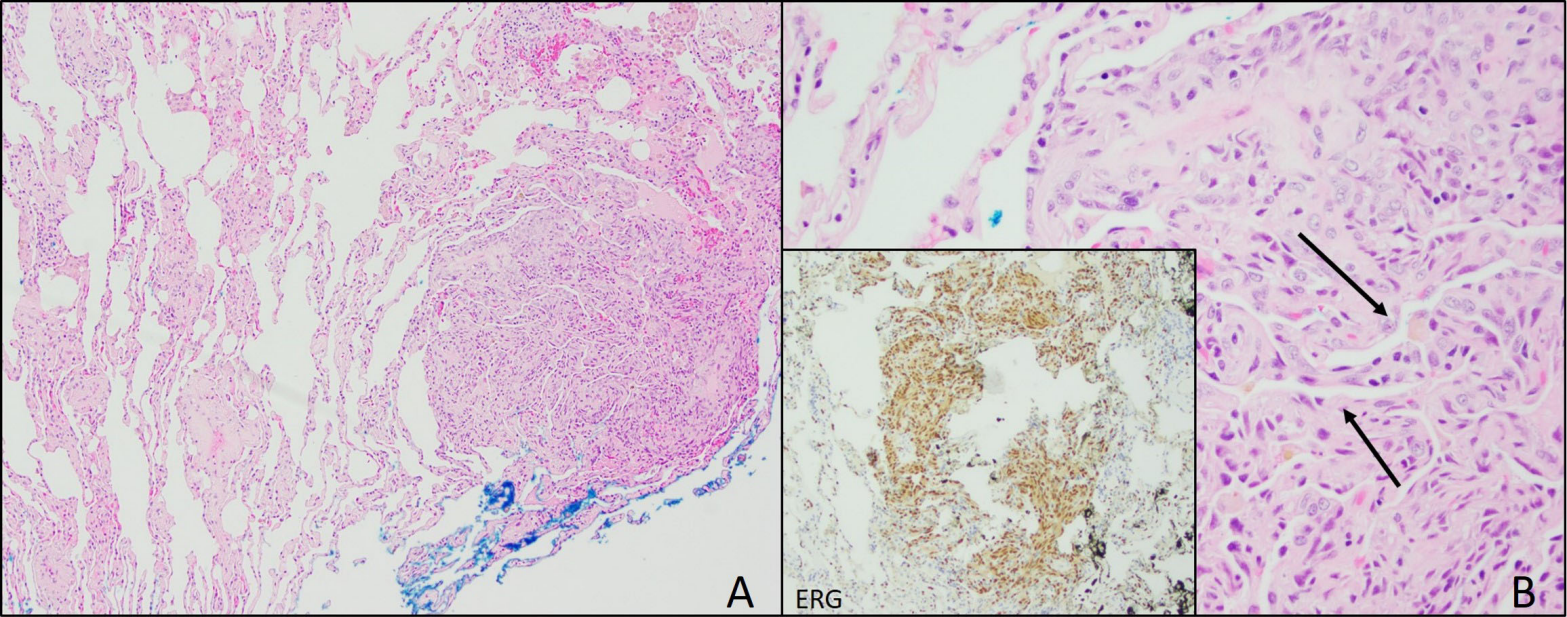
Lung Biopsy. **(A)** Lung biopsy from 2021 showing focal, well-circumscribed nodules comprised of atypical spindle cells. **(B)** Plump spindle cells showing slit-like vasoformative channels (arrows). Lung biopsy also showing ERG positive cells (inset) and CD31 (not pictured).

In June 2021, video-assisted thoracoscopic (VATS) lung biopsy of the right upper and middle lung lobes was performed due to worsened innumerable lung nodules first seen in December 2020 (**Figure 2A**). Histopathology results reported multiple nodular lesions primarily composed of spindled cells growing in short fascicles and forming slit-like lumina containing red blood cells (RBCs) and hemosiderin deposits in a focally fibrous background. IHC stains were positive for CD31, CD34, ERG, FLI-1, collagen IV, and D2-40 (**Figure 2B**).

In July 2021, BMBx revealed bone marrow diffusely involved by an atypical spindle cell proliferation with occasional polygonal cells, extensive osteosclerosis, markedly decreased hematopoiesis, and MF3. IHC stains were strongly positive for CD34, CD31, ERG, and FLI-1. Next-generation sequencing (NGS) showed *RUNX1* mutation, PI3K pathway mutation (i.e., PIK3CA H1047R), and low tumor mutation burden. NGS analysis was negative for *NRAS* variants. Unlike the paratrabecular fibrotic process seen in previous biopsies, these findings were consistent with a low-grade vasoformative neoplasm suggestive of KHE with likely multifocal disease to the lungs. Although previous bone marrow biopsies were considered to represent myelofibrosis, the histologically similar vasoformative spindle cell proliferation and immunostains favored a neoplastic process with endothelial differentiation in the bone marrow and the lung. Upon definitive diagnosis, baseline blood tests showed persistent macrocytic anemia (Hb = 7.4 g/dL, MCV = 121.2) and thrombocytopenia (50 × 10^3^/μL). Consumptive coagulopathy was absent. Hence, no evidence of KMP was seen. She was started on systemic steroids with daily prednisone 7 mg and pazopanib 400 mg; however, in March 2022, the patient showed evidence of progressive disease. We believed that our patient needed a more aggressive treatment because of the multifocal nature of her disease, so we started her on weekly paclitaxel for a total of seven doses; however, restaging showed mild progressive disease. Since the patient was having measurable progressive disease over a short interval of time, cytotoxic chemotherapy was used to eliminate proliferative clones within the tumor. Hence, in June 2022, she began gemcitabine and docetaxel at standard doses. Unfortunately, worsened thrombocytopenia and anemia were seen all along, so gemcitabine monotherapy was used and was stopped in September 2022, again due to progressive disease.

**Figure 3:**
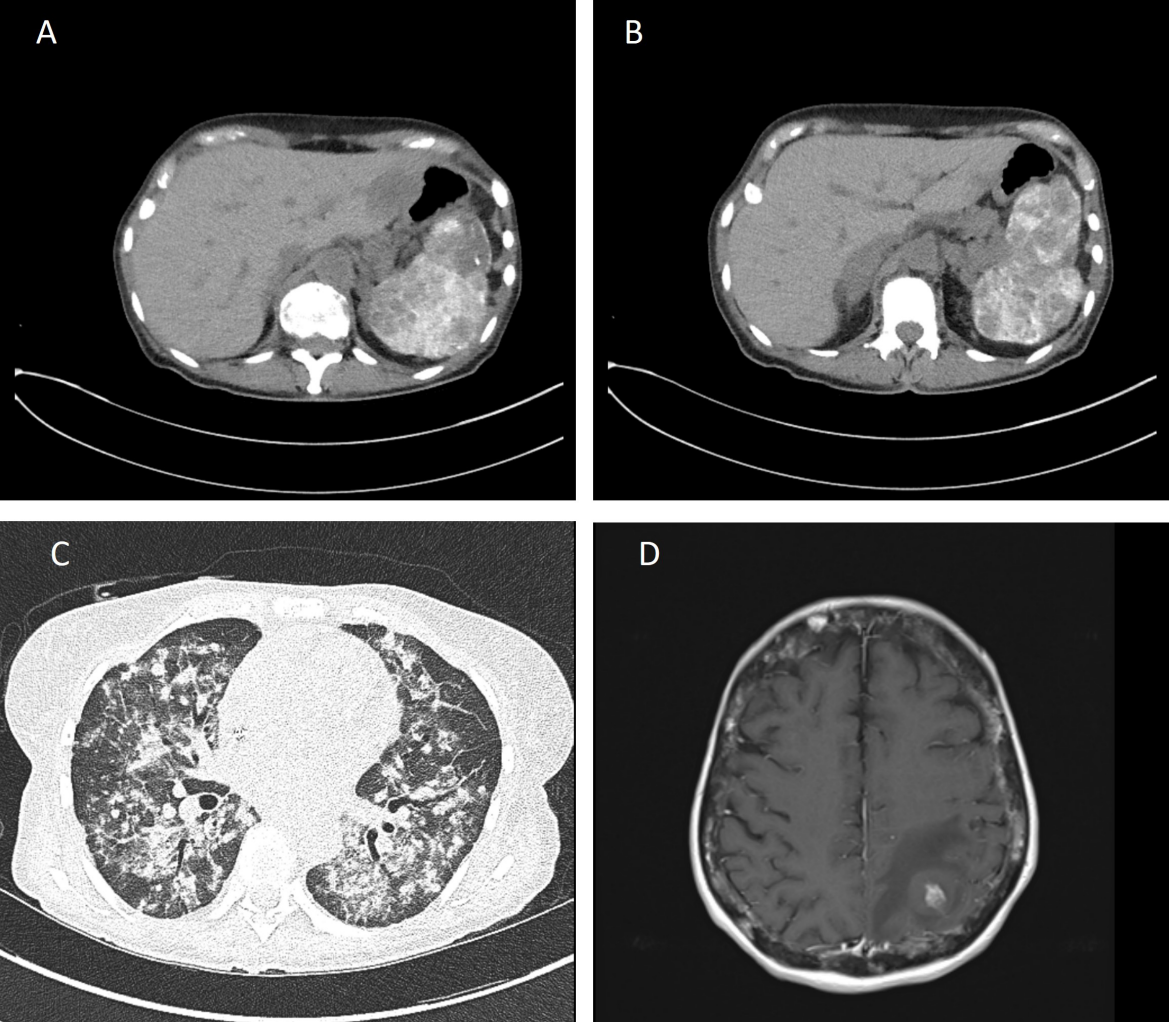
CT scans without contrast and MRI of the tumor lesions. **(A, B)** Upper left and right images of a CT scan of the abdomen and pelvis showing hypodense tumor lesions in the liver **(A)** and spleen **(B)**. **(C)** Lower left image showing a chest CT scan of the lung with innumerable pulmonary nodules seen throughout the lungs. **(D)** MRI of the brain showing five supratentorial lesions with minimal enhancement within bilateral cerebral hemispheres. The largest lesion found within the superior left parietal lobe measured 2.4 cm in maximal dimension. Findings consistent with multiple intracranial hemorrhagic metastases with surrounding vasogenic edema.

Up until this point, our patient experienced severe fatigue with declining performance status and dyspnea with any exertion. Based on her persistent anemia and thrombocytopenia, she became dependent on RBC transfusions since approximately September 2021 with the additional need of platelet transfusions since June 2022, both of which had been administered up to every 2 weeks until pembrolizumab was started in October 2022. Shortly after the initiation of pembrolizumab, her blood counts increased (Hb = 8.4 g/dL, platelets = 147 × 10^3^/μL) and she was platelet- and RBC transfusion-independent 2 months later, suggestive of bone marrow improvement. However, BMBx done on March 24^th^, 2023 still showed persistent cellular spindle cell proliferation and markedly decreased hematopoiesis. In addition, restaging CT scans and MRI were compatible with disease progression and new brain metastases accompanied by both motor and cognitive dysfunction, as well as worsening dyspnea on exertion (**Figure 3**). A month later, the patient died.

## Discussion

We hereby present a challenging case of KHE in an adult, replacing bone marrow tissue and secondary myelofibrosis with multifocal involvement in the lung, liver, and brain. KHE was initially described in children by Zukerberg et al. in 1993 (1). The first adult KHE cases were reported by Mentzel et al. in 1997 (3). However, these cases exhibited locally aggressive behavior, with intermediate malignant behavior and debatable multifocal potential (3, 28, 29). KHE is not known to exhibit distant metastasis, and whether it can be described as multifocal or metastatic when present in multiple locations is still a matter of debate (3, 4, 10, 11, 28).

**Figure 4:**
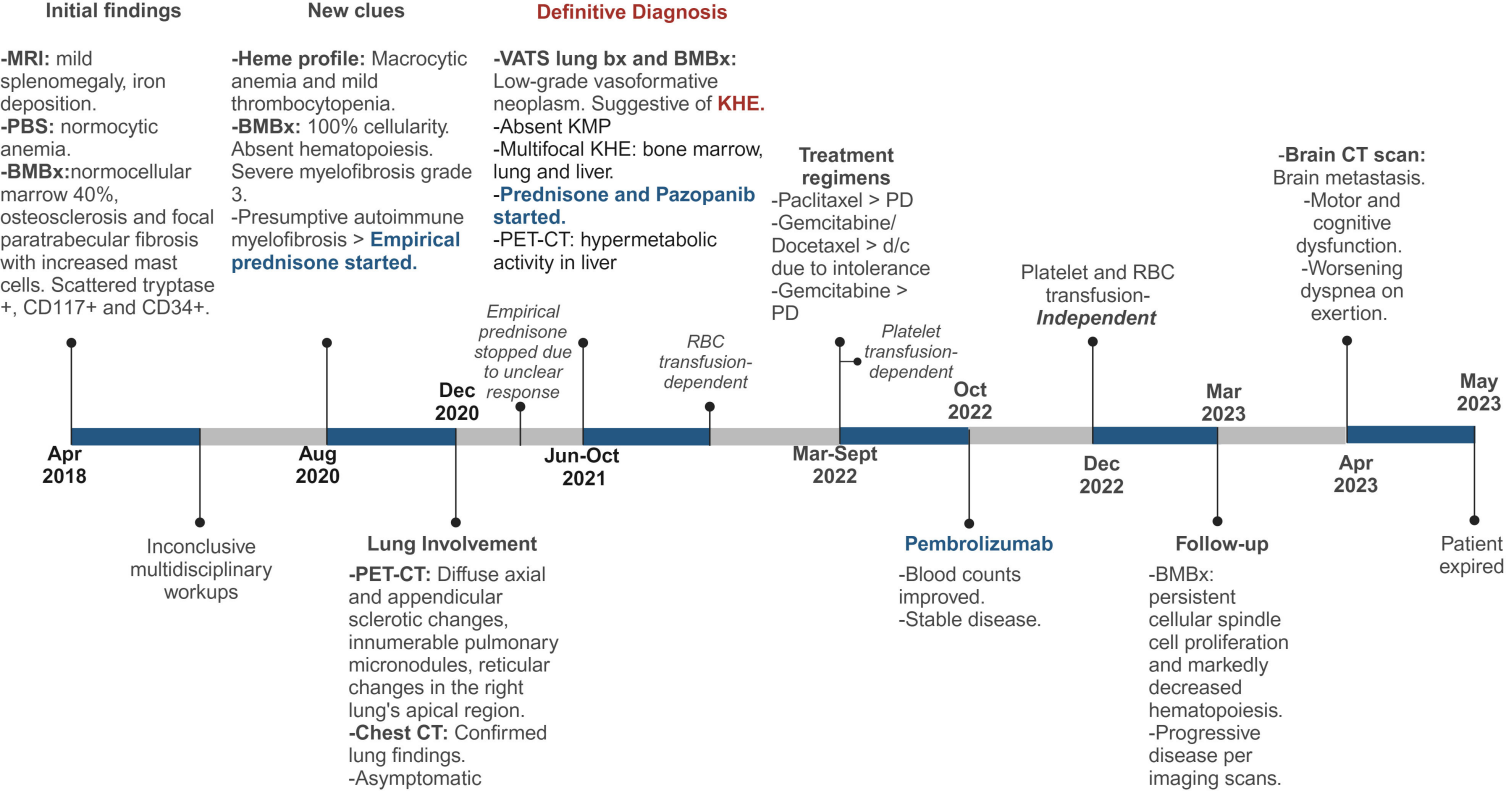
Detailed Timeline of Events. MRI, magnetic resonance imaging; PBS, peripheral blood smear; BMBx, bone marrow biopsy, PET-CT, positron emission tomography scan; CT, computed tomography; Bx, biopsy; KHE, kaposiform hemangioendothelioma; PD, progressive disease. Created with Biorender.com.

Bone marrow biopsies and a lung biopsy ultimately revealed KHE in our patient after three extensive years of workup (**Figure 4**). Multifocal KHE was our final diagnosis given the histological appearance, positive staining pattern for endothelial and lymphatic markers, and low-grade behavior. The initial presence of splenomegaly with underlying iron deposition and bone marrow fibrosis led to a primary impression of this being consistent with a myeloproliferative disorder. Subsequent workups ruled out POEMS syndrome (polyneuropathy, organomegaly, endocrinopathy, monoclonal plasma cell disorder, skin changes), Gaucher’s disease, diffuse osteosclerosis, plasma cell neoplasms, and myeloproliferative neoplasms as possible differential diagnoses. Kaposi sarcoma was considered as a differential diagnosis given the presence of spindle cell proliferation with small, slit-like vascular spaces, but was ruled out due to HHV-8 negativity. Kaposiform lymphangiomatosis (KLA) was also considered in the differential diagnosis since this lesion showed many of the same histopathological and clinical features as KHE, and differentiation between multifocal KHE and KLA can be difficult (30). However, molecular analysis of both entities demonstrated recurrent *NRAS* mutations in KLA and mutations of the PI3K pathway in KHE (31, 32). In this case, the histological finding of more defined nodules of spindle cells also favored the impression of KHE over KLA (30). Epithelioid hemangioendothelioma was also considered due to the indolent course and the presence of both spindled and occasional polygonal cells, but was ruled out given the negative fluorescence *in situ* hybridization (FISH) studies for both *WWTR1*–*CAMTA1* and *YAP1*–*TFE3* fusions. Lastly, angiosarcoma was also ruled out due to the lack of aggressive behavior and the clinical and histopathological characteristics of this case. The findings reported in our case are consistent with those previously reported in the literature (3, 11, 29), specifically with the findings reported by Azma et al. in a pulmonary KHE with involvement of the spleen and bone, which showed multiple foci of spindle-shaped cells with many individual small lumens containing RBCs and with positive IHC stains for CD31 and CD34 (11).

To date, there is no consensus regarding an ideal treatment of KHE. Most cases are managed based on expert opinion, review of available evidence, and clinical experience as the disease is rather rare and only a few prospective clinical trials have been done (7, 24, 27). The choice of treatment is often driven by several factors such as the extent of the tumor, location, accessibility to excision, vascular invasion, and the presence or absence of KMP (2, 7, 24). The combination of sirolimus with transarterial embolization was assessed in a retrospective study by Brill et al. in a small cohort of children with KHE and KMP (33). The authors reported a median resolution of KMP of 7 days *vs*. 3 months in patients with sirolimus alone. However, the tumor response and rebound KHE with KMP were not statistically significant between cohorts (33). Corticosteroids plus sirolimus have also been shown to be effective and safe in treating KHE accompanied by KMP (7, 9). In a Chinese randomized clinical trial, this combined regimen was found to have superior clinical benefits in improving the signs and symptoms of active KHE accompanied by KMP than sirolimus alone in pediatric patients. Patients in the combined therapy cohort showed durable platelet response, significant improvement in hypofibrinogenemia, lower rate of KMP rebound, reduced blood transfusions, and lower total incidence of disease sequelae compared with the monotherapy cohort (24). Based on results from the current literature, sirolimus has been shown to be effective and safe for complicated KHE by KMP and has rapidly been considered an optimal first-line therapy for this entity. However, prospective studies with a bigger sample size are still needed to validate these findings, as the exact duration of therapy and the dose of sirolimus in patients with KHE are yet to be established. Despite persistent severe thrombocytopenia, our patient never developed consumptive coagulopathy, hence no evidence of KMP. Sirolimus was never tried as part of our multidisciplinary approach as her disease was not complicated by KMP. This is an intriguing aspect of our case, which may be explained by the initial indolent nature of her disease marked by progressive bone marrow fibrosis. In addition, our patient was an adult and the secondary myelofibrosis accompanied by the overall behavior of this tumor made it challenging to suspect KHE clinically. Unclear response to multiple treatment lines was observed in our patient. Another fascinating factor of this case was seeing how her transfusion requirements resolved briefly with pembrolizumab to an extent that was not compatible with cessation of myelosuppressive chemotherapy. Notwithstanding the limitations of a single case report, this case could suggest that KHE may also present with secondary myelofibrosis in adults; however, further investigation is needed.

## Conclusion

KHE is a rare vascular neoplasm with aggressive behavior that occurs mainly in children. Although rare, reports in adults have been observed, and KHE should be considered as a differential diagnosis in this population. Histological findings, IHC, and imaging aid in distinguishing KHE from other vascular malignancies. Further prospective studies and case series with molecular profiling are needed to better understand its malignant and metastatic potential and to identify potential therapeutic options.

## Data availability statement

The original contributions presented in the study are included in the article/supplementary material. Further inquiries can be directed to the corresponding author.

## Ethics statement

Written informed consent was obtained from the individual(s) for the publication of any potentially identifiable images or data included in this article.

## Author contributions

AB: Methodology, Project administration, Visualization, Writing – original draft, Writing – review & editing. MA: Conceptualization, Data curation, Validation, Visualization, Writing – review & editing. AS: Writing – review & editing. ZY: Writing – review & editing. BB: Supervision, Validation, Writing – review & editing. VR: Funding acquisition, Supervision, Validation, Writing – original draft, Writing – review & editing.
